# Minimally invasive trans-sulcal parafascicular surgery for the early evacuation of spontaneous intracerebral hemorrhage: the ENRICH trial

**DOI:** 10.1007/s11739-025-03907-5

**Published:** 2025-03-03

**Authors:** Felice Cinque, Davide Nilo, Alessandro Gezzi, Fabrizio De Gregorio, Bianca Clerici, Scuola di metodologia della ricerca, SIMI

**Affiliations:** 1 S.C. Medicina Indirizzo Metabolico, Fondazione IRCCS Ca’ Granda Ospedale Maggiore Policlinico Di Milano, 20122 Milan, Italy; 2 Department of Pathophysiology and Transplantation, University of Milan, 20122 Milan, Italy; 3 Department of Advanced Medical and Surgical Sciences, University of Campania “Luigi Vanvitelli”, Naples, Italy; 4 Internal Medicine and Geriatrics, IRCCS INRCA, Via Della Montagnola, 81, 60127 Ancona, Italy; 5 Department of Clinical and Molecular Sciences, Università Politecnica Delle Marche, Ancona, Italy; 6 S.C. Medicina Generale II, Ospedale San Paolo, ASST Santi Paolo e Carlo, Dipartimento di Scienze della Salute, University of Milan, Via A. di Rudinì 8, 20142 Milano, Italy; 7 Dipartimento di Scienze Biomediche, Chirurgiche ed Odontoiatriche, University of Milan, Milano, Italy

## Background

Spontaneous intracerebral hemorrhage (ICH) is associated with a substantial 40% mortality rate within 1 month and significant morbidity, with only 10–25% of patients recovering functional independence [1]. According to current guidelines, patients with supratentorial ICH volumes of 20–30 ml and a Glasgow Coma Scale (GCS) score in the moderate range (5–12) may benefit from minimally invasive surgical options in terms of reduced mortality [2]. However, there is no consensus on the efficacy of any surgical technique in improving functional outcomes, as results from large randomized clinical trials remain inconclusive. Among the various techniques available, minimally invasive trans-sulcal parafascicular surgery (MIPS) has demonstrated safety, prevention of re-bleeding, and maximized clot evacuation in preliminary studies [3, 4]. The "Early Minimally Invasive Removal of Intracerebral Hemorrhage" (ENRICH) trial was designed to compare the outcomes of MIPS along with medical management and medical management alone in patients with spontaneous supratentorial acute ICH.

## Summary

The ENRICH trial is a prospective, multicenter, adaptive, randomized clinical trial conducted from December 1^st^, 2016, to August 24^th^, 2022, at 37 centers in the USA [5]. The trial enrolled patients aged 18–80 years with computed tomography (CT)-confirmed supratentorial, spontaneous acute ICH. Inclusion criteria encompassed a hematoma volume of 30–80 ml, GCS of 5–14, a National Institutes of Health (NIH) stroke scale over 5, a pre-ICH modified Rankin scale of 0–1, and the possibility to start surgery within 24 hours since symptom onset. Exclusion criteria included uncorrectable coagulopathy, need for long-term anticoagulation, intraventricular hemorrhage over 50% of either lateral ventricle, primary thalamic or infratentorial hemorrhage, or a secondary cause of ICH. Patients were randomized 1:1 to MIPS plus guideline-based medical management (surgery group) or guideline-based medical management alone (control group). The primary efficacy end point was the utility-weighted modified Rankin scale score (ranging from 0, no symptoms, to 6, death) at 180 days. Primary safety end points included 30-day mortality and change in hematoma volume from the initial to the follow-up neuroimaging. Pre-specified secondary end points were postoperative rebleeding associated with neurologic deterioration, length of stay in the intensive care unit, length of stay in hospital, modified Rankin scale ordinal score at 7 days or at discharge (whichever came first) and at 30, 90, and 180 days, change in hematoma volume after surgery completion, hematoma volume after surgery completion, modified Rankin scale dichotomized score at 180 days, and overall survival at 180 days. Patients and providers were not blinded to the trial group. However, the modified Rankin scale interview was audio-recorded and reviewed by an independent neuropsychologist unaware of treatment allocation. Neuroimaging was centrally adjudicated by two neuroradiologists, one of whom was unaware of treatment allocation. The study enrolled 300 patients, 150 in each group. The median age was 64 years (interquartile range, 56–72) in the surgery group and 62 years (interquartile range, 51–73) in the control group. Females comprised 48% of the surgery group and 52% of the control group. Most enrolled patients had lobar hemorrhage (69.3%) because enrollment of patients with anterior basal ganglia hemorrhage was stopped for futility after the second interim analysis. The mean utility-weighted modified Rankin scale score at 180 days was 0.458 in the surgery group and 0.374 in the control group, with a between-group difference of 0.084 (95% Bayesian credible interval, 0.005–0.163; posterior probability of superiority of surgery, 0.981). In lobar hemorrhages, the between-group difference was 0.127 (95% Bayesian credible interval, 0.035–0.219), while in anterior basal ganglia hemorrhages it was −0.013 (95% Bayesian credible interval −0.147 to 0.016). The 30-day mortality rate was 9.3% in the surgery group and 18.0% in the control group (estimated difference: −8.7, 95% Bayesian credible interval −16.4 to −1.0; posterior probability of superiority, 0.987). Postoperative rebleeding associated with neurologic deterioration occurred in 3.3% of surgery patients. The authors concluded that early minimally invasive hematoma evacuation by MIPS along with guideline-based medical management led to better 180-day functional outcomes compared to medical management alone. Such difference was attributed to the intervention for lobar hemorrhages.

## Strengths of the study

- The study addresses a clinically relevant question.
- The ENRICH trial involved 37 centers, thus enhancing the external validity of the study.
- Due to the technical impossibility of blinding patients undergoing surgical treatment, the authors resorted to blinded, independent adjudication of pivotal study end points (e.g., modified Rankin scale interviews, neuroimaging data). This approach effectively mitigates the lack of blinding of patients and providers involved in the ENRICH study.

## Weaknesses of the study

- Although the study protocol allowed the inclusion of a broad age range of participants, the population enrolled in this study was highly selected both in terms of etiological criteria, excluding all secondary causes of acute ICH, and severity criteria, including only patients with a hematoma volume between 30 and 80 ml and mild-to-severe neurological deficits. This might reduce the generalizability of the study results and limit their clinical applicability to unselected ICH inpatients.
- Because the study was designed to compare MIPS and guideline-based medical management, the trial offers no insight into the performance of MIPS compared to other surgical approaches.

## Question marks

Although the authors emphasized the applicability of the trial findings only to the selected study population, we are left wondering if MIPS can potentially be employed also for cases of spontaneous ICH at different anatomical locations and/or of greater severity and for the treatment of secondary ICH.

## Clinical bottom line

In this study on early evacuation of acute spontaneous supratentorial ICH, MIPS along with medical treatment resulted in a better functional outcome at 180 days compared to medical treatment alone. Although the study population was highly selected and poses a question on the external validity of the study results, MIPS appears to be the treatment of choice for a clinical condition—spontaneous ICH—which currently entails significant morbidity and mortality. Its applicability to other clinical scenarios and patient subsets deserves further investigation.

## Data Availability

Not applicable.
